# Application of the extended theory of planned behavior to predict dentist intention and behavior in providing caries preventive care for preschool children

**DOI:** 10.1186/s12903-023-03694-5

**Published:** 2023-12-06

**Authors:** Safira Khairinisa, Risqa Rina Darwita, Diah Ayu Maharani, Febriana Setiawati

**Affiliations:** Department of Preventive and Public Health Dentistry, Faculty of Dentistry, University of Indonesia, Jakarta, 10430 Indonesia

**Keywords:** Dentists, Preventive dentistry, Early childhood caries, Theory of planned behavior, Structural equation modelling

## Abstract

**Background:**

Various studies show a gap between evidence-based recommendations and the preventive practice of dentists. This study aimed to create and assess an extended Theory of Planned Behavior (TPB) model on dentists' intentions to do caries preventive measures and related barriers experienced by dentists.

**Methods:**

A total of 362 general dentists from 34 Indonesian provinces were included in this study. A self-administered online questionnaire consisted of three sections: dentist characteristics and practice pattern, TPB questionnaire, and dentist perceived behavior regarding preventive care for pediatric patients. The questionnaire was distributed and the data was analyzed through structural equation modeling.

**Results:**

TPB's extended version is a fit and relevant model, explaining 55.3% of dentists' intentions to undertake preventive procedures and 17.8% of preventive practices. Perceived behavioral control was the most powerful predictor of intention (44.2%) and practice (8.8%), while parental barriers were the most significant barrier to provide preventive care (18.9%).

**Conclusion:**

Extending the TPB by taking barriers from multiple stakeholders as a consideration has a higher predictive level for preventive practices. Each barrier should be addressed through oral health programs and policies, and dentists must be taught to overcome these barriers (through formal or continuing education) in order to maximize caries prevention strategies.

## Background

Oral diseases in children continue to be a major global public health issue, with severe negative consequences for quality of life [1]. The presence of one or more decaying (noncavitated or cavitated lesions), missing (due to caries), or filled tooth surfaces in any primary tooth in a child aged 71 months or younger is described as early childhood caries (ECC) [2]. ECC affects 90.2% of 5-year-old children in Indonesia, making it one of the countries with the highest rates [3].

The high prevalence and large burden of disease from dental caries, especially in young populations, urge caregivers and stakeholders to solve the problem [3–5]. In clinical settings, dentists provide preventive services such as dental examinations for children, caries risk assessments, oral health counseling for parents, and fluoride agent applications [6]. Various studies show that there is a gap between evidence-based dentistry and the practice of dentists [7, 8]. Even though dentists already understand the benefits of preventive services, many factors hinder their implementation [7, 9, 10]. This gap can occur due to determinants such as attitudes, beliefs, and values regarding interests and the ease of taking preventive measures [11, 12]. An earlier meta-analysis identified the theory of planned behavior (TPB) as potentially the most valid behavior change theory for predicting, characterizing, and comprehending oral health habits [13]. Based on this theory, the strength of this intention is influenced by three constructs: attitude toward the behavior, perception of personal control over the behavior, and belief in a subjective norm [14, 15]. Yusuf (2016) applied TPB and found that attitude was the most important predictor of dentists' provision of preventive practices such as education on diet, smoking, and alcohol [7]. Bonetti (2010) shows that all constructs in TPB predicted the application of pit and fissure sealants by Scotland's dentists [16].

One of the limitations of TPB is that the construct does not consider whether there are opportunities and resources to carry out the desired behavior regardless of intention [17]. However, TPB is a flexible model that allows for the addition of variables that can enhance the explained variance [13]. Some previous studies extended the TPB original version and added a few other variables, such as entrepreneurial situational factors, perceived threats, health knowledge, and past behavior [17–19]. The Integrative Behavioral Model (IBM), which incorporates components from TPB, introduces modified determinants that influence behavior intention, such as attitude, descriptive norm, personal agency, self-efficacy, knowledge and skills, salience, environmental constraints, and habit [19, 20]. Through this study, internal and external barriers that dentists experience as a result of the difficulties of providing dental care to young children should also be investigated [9, 21–23].

As one of the important factors, dentists' behavior and barriers in treating children, especially in terms of preventive care, need to be considered to prevent more complex dental and oral health problems when children grow up [23]. However, multiple studies showed that there are knowledge-practice gap in providing preventive care among dentists [8, 11, 16, 24]. In order to increase the efficiency of dental visits and the early utilization of caries preventive treatment, it is important to understand the determinants and barriers that prevent clinicians from administering such treatment to preschoolers. Therefore, the present study aimed to develop and assess an extended TPB model on dentists' intentions and practice regarding caries preventive measures in pediatric patients and the associated barriers, which may provide preliminary clues for future interventions in preventive practice and the oral healthcare system.

## Methods

This study was conducted in November 2022 to explore dentist attitudes, subjective norms, perceived behavior controls, and barriers regarding ECC prevention in a clinical setting in Indonesia. This study was reviewed and approved by the ethics committee of the Faculty of Dentistry, Universitas Indonesia (protocol No. 031101022). The sample size was calculated using G*Power v.3.1.1 (A.gpower.hhu.de) and found that a minimum of 105 dentists from each group is required to have a significance of 0.05 and a power of 95%, assuming an effect size of 0.25 [10, 11]. Taking into consideration possible selection bias, 350 dentists were set as the minimum sample in this study. Snowball sampling was used to recruit general dentists across the country. The inclusion criteria for participants were general dentists who were willing to participate and had at least one year of practice. Specialists were excluded from this study. 34 key-person were recruited for each of the 34 provinces in Indonesia, who then distributed the online questionnaire to general dentists in their working region.

Participants who agree to participate in this study complete a self-administered questionnaire using Google Forms that were shared online through social media (WhatsApp or Instagram) by the key person in each group. Before proceeding to the main part of the questionnaire, participants were given an explanation of the study's objectives and given their informed consent. A self-administered online questionnaire consisted of three sections: dentist characteristics and practice pattern (10 items), TPB questionnaire (12 items), and dentist perceived behavior regarding providing preventive care for pediatric patients (20 items). Dentists' characteristics and practice patterns were measured using the questionnaire listed in Table 1. The TPB questionnaire was adapted from a previously used questionnaire by changing the object of preventive care specifically to preventive care for children under six years old [7]. The perceived barrier questionnaire was made by combining previous studies that emphasize barriers in early childhood treatment and barriers in preventive treatment questionnaires [21, 22, 25]. This present study only included relevant items with the study objectives that went through a cross-cultural adaptation process [26]. The original questionnaire was made into an Indonesian version by professional translators. Then, it was assessed and sorted for its relevancy to the aim of this study,and adjusted by literature reviews. An expert panel made up of three dental public health specialists looked over the Indonesian versions that were put together into a fixed translation. Back translations were done by professional translators independently. Then, the translated and back-translated versions were looked over by experts, who made a combined back-translated version. An assessment was made of whether the adapted questionnaire items were comparable to the original ones [26, 27].

**Table 1:** Dentists' practice pattern

Variables	Details
**Practice Years**	Long time respondents practiced since graduating to become a dentist to the present
**Practice Sector**	Respondents could choose more than one answer regarding their practice place: primary health care, government hospitals, private hospitals, private practices, or BPJS (Indonesian National Health Coverage) clinic. In conducting an analysis, it will be re-categorized again as the government (government hospitals, primary health care facilities, BPJS clinic), private (private hospitals, private practices), or a combination of both
**Weekly Working Hours**	Respondents were asked to estimate their weekly working hours as dentists
**Weekly Patient**	Respondents were asked to estimate their weekly patients number
**%Pediatric Patients**	%Paediatric Patients calculated by dividing average number of pediatric patients by the number of estimated their weekly patients
**%Preventive Pediatric Patients**	%Preventive Pediatric Patients calculated by dividing the average number of pediatric patients who receive preventive treatment/week by the number of estimated their weekly pediatric patients
**% Insuranced Patients**	Respondents were asked about the estimates the percentage of patients who pay using both private or government insurance compared to all patients who are treated
**Preventive Practice**	Respondents were asked to estimate the number of each preventive practice (oral hygiene and diet education [**EDU**], caries risk assessment [**CRA**] topical application fluoride [**TAF**] and silver diamine fluoride applications [**SDF**]) conducted in the past month

Both adapted questionnaires were validated through pilot testing on 42 general dentists, and all items are shown in Table 2. A pilot study was conducted to assess the validity, reliability, and acceptability of the questionnaire. A reliability test was conducted during the pilot study to measure internal consistency and external consistency. Internal consistency is measured using Cronbach's alpha and corrected item-total correlation (CITC) (Cronbach’s alpha = 0.886 and 0.887; CITC > 0.3). External constructs are measured using the interclass correlation coefficient (ICC) through test–retest in a 2-week interval (ICC = 0.888 and 0.886). Participants answered a five-point Likert scale ranging from 0 = ‘strongly disagree' to 4 = ‘strongly agree' in the TPB and perceived barrier questionnaire.

**Table 2:** Variables regarding the theory of planned behavior and dentists' perceived barrier to provide dental caries preventive practices

Domain	Question
**Attitude**	1. Dentists must provide education on diet and toothbrushing to (parents) preschool children (**A1**) 2. Dentists must examine risk factors for preschool children (**A2**) 3. Dentists should recommend the application of fluoride to preschool children, if indicated (**A3**) 4. Dentists should recommend the application of silver diamine fluoride to preschool children, if indicated (**A4**)
**Subjective Norm**	1. Most dental colleagues provide caries preventive care to all preschool children (**SN1**) 2. All my patients (parents) think that as a dentist, I have to provide their children with preventive care (**SN2**)
**Perceived Behavior Control**	1. I am confident that I can provide caries prevention services to preschoolers (**PBC1**) 2. For me, providing caries preventive services to preschoolers is very easy (**PBC2**)
**Intention**	1. I intend to conduct education on diet and tooth brushing to (parents) all of my preschool age patients (**I1**) 2. I intend to examine caries risk factors for all my preschool age patients (**I2**) 3. I intend to apply topical fluoride to all of my preschool age patients if indicated (**I3**) 4. I intend to apply silver diamine fluoride to all of my preschool age patients if indicated (**I4**)
**Child-Related Barrier**	1. Child patient who gets angry / cries easily when receiving dental treatment (**C1**) 2. Pediatric patients do not cope well with dental treatment (**C2**) 3. Child patients do not like to sit in the dental unit (**C3**) 4. Most pediatric patients are afraid of dental treatment (**C4**) 5. The condition of the teeth and mouth of pediatric patients who come to the dentist is too bad to be given preventive treatment (**C5**)
**Dentist-Related Barrier**	1. Preventive dental care has a low priority over other treatments **(D1)** 2. Preventive dentistry is not highly prioritized in the dental education curriculum (**D2**) 3.Preventive materials are not available where I work because I did not ask for them (**D3**) 4. Preventive dental care is not profitable for dentists (**D4**) 5. In my opinion, dental care for children causes stress (**D5**)
**Parents-Related Barrier**	1. Parents have less knowledge about caries preventive measures (**P1**) 2. Parents tend not to want dentists to treat their children's teeth. (**P2**) 3. Parents think primary tooth is unnecessary (**P3**) 4. preventive dentistry considered unnecessary (**P4**) 5. Parents ignore routine visits to the dentist if there are no complaints (**P5**)
**Health Care System-Related Barrier**	1. Public/private insurance does not cover the cost of preventive dental treatment (**HC1**) 2. Materials for preventive dentistry are not available where I work (**HC2**) 3. The payment I receive for preventive dental care for my child is inadequate (**HC3**) 4. Dental care for children is currently more focused on curative than preventive (**HC4**) 5. The dental health service system in Indonesia for preschool-age children is not good enough (**HC5**)

### Data analysis

SPSS 23 software (IBM Corp., Armonk, NY, USA) was used for descriptive analysis to analyze the means and standard deviation (SD) for numerical variables and the prevalence for categorical variables. Partial least squares (PLS) modeling using SmartPLS 3.2.9 was used for multivariate analysis by assessing the association between multiple variables to predict dentist intention and preventive practice simultaneously. PLS is a multivariate linear regression model method that detects correlations between matrices of independent and covarying descriptors and response variables [28, 29]. The coefficient of determination (R^2^) was interpreted as the proportion of the variance in the dependent variable that is predicted by the independent variable. The path analysis (β) determined the causal linkage between exogenous and endogenous variables using bootstrapping (*p* value < 0.05) [29].

## Results

**Fig. 1:**
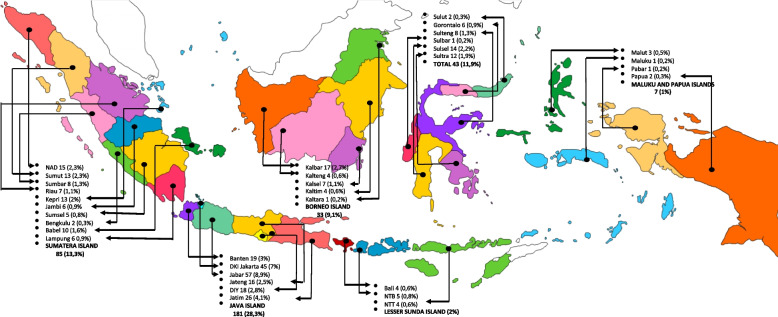
Distribution of the Respondents across 34 Province of Indonesia (*n*=362)

This study examined data from 362 general dentists from 34 Indonesian provinces (Fig. 1). With an average age of 32.8 years and 6.7 years of work experience, the majority of respondents (80.4%) were female. Dentists in this study worked in a range of settings, with 38.4% working entirely in the private sector, working 27.5 h per week, and seeing an average of 27 patients per week. However, of the entire number of patients, only 5–6 pediatric patients receive two preventive treatments every week. When compared to the total number of patients treated, 23.3% were pediatric patients, and 41% of them received preventive care assessed in this study. 77.9% of respondents work at a health care facility that offers insurance services, with an average of 34.6% of their patients who are served using these services. Details on dentists' characteristics and practice patterns are shown in Table 3.

**Table 3:** Dentists' Characteristic and Practice Pattern (*n*=362)

Dentists' Characteristic	n (%)	x (SD)
**Sex**		
Male	71 (19.6%)	
Female	291 (80.4%)	
**Age (years old)**		32.8 (8.2)
**Practice Years**		6.7 (6.5)
< 10 years	257 (71%)	
≥ 10 years	105 (29%)	
**Practice Sector**		
Private	139 (38.4%)	
Government	88 (24.3%)	
Both	135 (37.3%)	
**Weekly Working Hours**		27.5 (16)
**Weekly Patients Number**		26.9 (19.9)
**%Pediatric Patients**		23.3 (16.7)
**%Preventive Pediatric Patients**		41 (37.2)
**% Insuranced Patients**		34.6 (36.3)

**Fig. 2:**
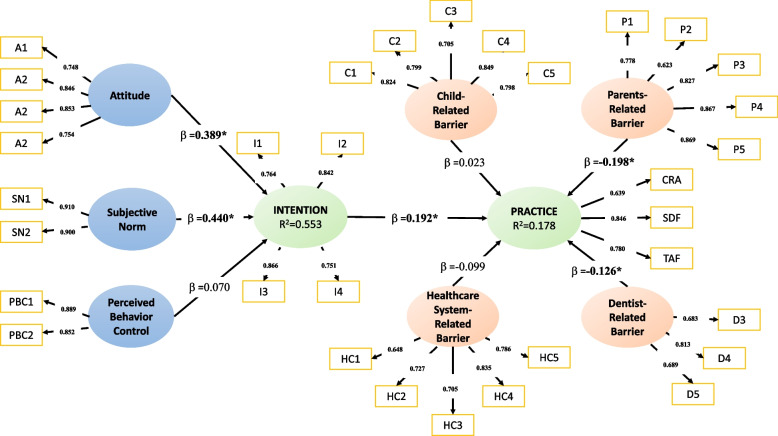
Research Framework of the Final (2^nd^) Model. Outer Model = Factor Loading value of exogenous variables; Inner Model = Path coefficient value (β) of endogenous variables; Construct = Overall model determination coefficient (R^2^)

The conceptual framework according to the research hypothesis was created in SmartPLS software to assess the TPB construct variables and the barriers experienced by dentists in carrying out caries preventive practices. The simulation of the PLS model was carried out by calculating and assessing various parameters, including the value of item loading, reliability, and validity. This involved a two-step process of calculating the PLS model parameters separately by completing the measurement model blocks (measurement model evaluation/outer model) and then estimating the path coefficients of the structural model (structural model evaluation/inner model). Finally, the overall model was validated by a power analysis test and determining the magnitude of the coefficient of determination [29]. After several model experiments, the model in Fig. 2 is the final model of the study.

The evaluation of the measurement model aims to evaluate the consistency and validity of the manifest variables (Table 4). Consistency evaluation is done through individual manifest and construct reliability tests. Variable validity is tested based on convergent and discriminant validity, while individual manifest reliability explains individual manifest variance relative to the latent variable by calculating the standardized outer loading of the manifest variable. Convergent validity refers to the principle that the manifest variables of a construct should be highly correlated. Based on the parameter value, indicators with an outer loading < 50 should be discarded, 0.50–0.70 can be retained by evaluating the AVE value, and > 0.70 is the ideal value [28, 29]. For variables with outer loading values between 0.50 and 0.70, the evaluation is based on whether the elimination of these indicators improves composite reliability. Average Variance Extracted (AVE) determines the amount of variance captured by the latent variable from its relative manifest variables due to measurement error. The recommended AVE value should be greater than 0.50, which means > 50% of the variance of the indicator can be explained [28].

**Table 4:** Results of measurement model evaluation (*n*=362)

Research Construct	Code	Scale	Model 1				Model 2				
		Mean (SD)	Alpha^a^	CR^b^	AVE^c^	Factor Loading^d^	Alpha^a^	CR^b^	AVE^c^	Factor Loading^d^	VIF^e^
Attitude	A1	3.72 (0.49)	0.814	0.878	0.643	0.748	0.814	0.878	0.643	0.748	1.705
	A2	3.49 (0.61)				0.846				0.846	2.065
	A3	3.48 (0.60)				0.853				0.853	2.046
	A4	3.06 (0.77)				0.754				0.754	1.454
Subjective Norm	SN1	2.86 (0.84)	0.776	0.900	0.819	0.910	0.776	0.900	0.819	0.910	
	SN2	2.73 (0.91)				0.900				0.900	
Perceived Behavior Control	PBC1	3.21 (0.64)	0.683	0.863	0.759	0.889	0.683	0.863	0.759	0.889	1.368
	PBC2	2.65 (0.88)				0.852				0.852	1.368
Intention	I1	3.42 (0.64)	0.820	0.882	0.651	0.764	0.820	0.882	0.651	0.764	1.644
	I2	3.15 (0.70)				0.842				0.842	1.924
	I3	3.17 (0.67)				0.866				0.866	2.254
	I4	2.84 (0.78)				0.751				0.751	1.714
Child-related Barrier	C1	2.28 (1.32)	0.859	0.897	0.635	0.822	0.859	0.897	0.635	0.824	2.183
	C2	1.88 (1.31)				0.804				0.799	2.153
	C3	1.55 (1.33)				0.713				0.705	1.701
	C4	2.30 (1.34)				0.849				0.849	2.204
	C5	2.39 (1.42)				0.792				0.798	1.619
Parents-related Barrier	P1	0.39 (0.89)	0.854	9.896	0.637	0.776	0.854	9.896	0.637	0.778	1.908
	P2	0.87 (1.24)				0.626				0.623	1.418
	P3	1.39 (1.44)				0.826				0.827	2.212
	P4	1.88 (1.46)				0.864				0.867	2.537
	P5	1.09 (1.30)				0.872				0.869	2.677
Dentist-related Barrier	D1	2.80 (1.13)	0.609	0.753	0.386	0.474	0.563	0.773	0.534	omitted	-
	D2	1.11 (1.31)				0.506				omitted	-
	D3	2.01 (1.37)				0.616				0.683	1.193
	D4	2.22 (1.35)				0.785				0.813	1.251
	D5	2.69 (1.26)				0.674				0.689	1.113
Health Care System-related Barrier	HC1	2.30 (1.31)	0.798	0.858	0.550	0.647	0.798	0.858	0.550	0.648	1.349
	HC2	1.99 (1.54)				0.715				0.727	1.462
	HC3	1.64 (1.40)				0.691				0.705	1.528
	HC4	2.69 (1.24)				0.849				0.835	1.861
	HC5	2.31 (1.33)				0.790				0.786	1.765
Preventive Practice	EDU	10.7 (16.8)	0.650	0.783	0.481	0.488	0.630	0.802	0.577	omitted	-
	CRA	5.1 (7.7)				0.707				0.639	1.147
	TAF	1.3 (2.7)				0.801				0.846	1.399
	SDF	0.3 (1)				0.737				0.780	1.327

Cronbach Alpha (a) and Composite Reliability (b) show the results of the reliability test. Cronbach Alpha > 0.6 and Composite Reliability > 0.7 are acceptable. Validity is assessed through the Average Variance Extracted (AVE) (c) and factor loading (d) score. Multicollinearity test is assessed through the Variance Inflating Factor (VIF) score (e); there is no multicollinearity if VIF < 5

The second parameter for consistency evaluation is structured reliability, which is evaluated by two measures, namely Cronbach's alpha and composite reliability (CR). Cronbach's alpha and CR indicate how well a set of manifest variables assesses a latent construct. However, compared to Cronbach alpha, composite reliability is considered a better measure of internal consistency as it uses the standardized loadings of the manifest variables. Nonetheless, the interpretation of composite reliability and Cronbach's alpha scores are the same. Research suggests that the Cronbach alpha value should be higher than 0.7, so all variables in this study already have good reliability [29].

In model 1, the variables D1, D2, and EDU have factor loading values that need to be evaluated. The AVE value in the domain of barriers experienced by dentists and preventive practices carried out shows poor convergent validity. Therefore, items D1, D2, and EDU were removed. In the final model, it can be seen that all variables have AVE values that meet the requirements and show good convergent validity.

Structural model evaluation is carried out to assess the relationship between exogenous latent variables through the path coefficient value (β) and the coefficient of determination (R^2^). The R^2^ value indicates the level of variance explained by the endogenous latent variable, while β indicates the strength of the influence from the variable to the endogenous latent variable [29]. In the final model of this study, the R^2^ value for the intention variable is 0.555. Therefore, attitude variables, perceived behavioral control, and subjective norms have a substantial degree that can explain the dentist's intention to carry out preventive practices. Based on the Theory of Planned Behavior, intention is the main predictor of preventive practice. However, this study shows that the R^2^ value for the preventive practice variables when only based on the intention predictor is only 7% for the TPB original construct. Based on the research hypothesis, adding barriers into consideration will increase the predictor level of preventive practice. When the variables of barriers from the child, parents, dentist, and health service system were added, could explain the preventive practice variable by 17.8%.

Beside R^2^, the path coefficients of all latent variables were compared to see which exogenous variables had the strongest influence on endogenous variables through non-parametric bootstrapping techniques. In this study, the bootstrapping test was conducted on 5000 samples, with the results shown in Table 5. The bootstrapping test shows that the exogenous variables of perceived behavioral control and attitude have t-values > 2.58 (*p* < 0.01) on the endogenous variables of intention and preventive practice. Also, barriers from parents and dentists also have a t-value > 2.58, so these variables also have significant effects on preventive practices carried out by dentists. The endogenous variable with the highest t-value is perceived behavioral control (t = 8.730; t = 3.657), means it has the most influences both to the intention and preventive practices carried out by dentists. The value of the path coefficient indicates the amount of endogenous variables affecting exogenous variables partially [29]. It can be seen in Table 5 that of the several endogenous variables that have a significant effect on exogenous variables, the attitude variable and perceived behavioral control have an effect of 38.9% and 44.2% on intention, as well as 7.7% and 8.8% to preventive practices. The Standardized Root Mean Square Residual (SRMR) value below 0.08 indicates that the model is fit, and Q^2^ > 0 shows that this model has predictive relevance [28, 29].

**Table 5:** Results of structural model evaluation

Path	Intention			Preventive Practice		
	Path Coefficient (β)^a^	t- value^b^	*p*-value	Path Coefficient (β)^a^	t- value^b^	*p*-value
Attitude	**0.389**	8.662*	< 0.001	**0.077**	3.657*	< 0.001
Perceived Behavior Control	**0.440**	8.730*	< 0.001	**0.088**	3.657*	< 0.001
Subjective Norm	0.072	1.545	0.123	0.015	1.405	0.161
Intention				**0.192**	4.180*	< 0.001
Child-Related Barrier				0.023	0.480	0.631
Parents-Related Barrier				**-0.189**	3.087*	0.002
Dentist-Related Barrier				**-0.126**	3.121*	0.002
Health Care System-Related Barrier				-0.099	1.694	0.091
**R**^**2c**^	**0.553**			**0.178**		
**Q**^**2**d^	**0.354**			**0.090**		
**SRMR**^e^	**0.068**					

^a^The path coefficient shows the magnitude of the direct and indirect effects of exogenous variables on endogenous variables

^b^ The t-value measures whether exogenous variables have a significant effect on endogenous variables (significant at t > 1.68 and *p* < 0.05)

^c^The Coefficient of Determination (R^2^) shows how much of the total variance of the construct is explained by the model

^d^The Q^2^ test shows the level of predictive relevance of the model (0.02–0.15: Low; 0.15–0.35: Moderate; > 0.35 High)

^e^The Model Fit test assesses how good the model under study is by Standardized Mean Square Residual value (Model Fit if SRMR < 0.08)

## Discussion

The main objective of this study is to determine the relationship between the variables in the TPB construct and the barriers experienced by dentists through a fit and relevant model. Theory of Planned Behavior limitation that it does not consider the existence of both internal and external barriers in predicting preventive practices became the background for this study [14]. This study continues to investigate the extent to which the original variables of the original TPB theory are related to intention (direct effect) and practice (indirect effect), along with the addition of barrier variables. In this study, the TPB model constructs, namely subjective norm attitudes and perceived behavioral control, can explain 54.9% of dentists' intention to perform preventive practices but only 7.6% of preventive practices performed. This is consistent with earlier research that found a disconnect between dentists' intentions and actual practice [7, 16, 30]. Despite not evaluating the same preventive procedures, Bonetti (2010) found that TPB construction might explain 30% of dentists' intention to perform pit and fissure sealants [16]. Although the degree of prediction varies according to the preventive measures assessed and the items used to explain the attitude, perceived behavioral control, and subjective norm, this study demonstrates that existing constructs are linear with theory-based predictions [16, 19].

Dentists, as health care providers, are unable to provide dental treatments without their patients' consent and willingness to pay [23]. Willingness to pay refers to the maximum amount in monetary terms that a patient is willing to sacrifice to obtain the benefits of a service [31]. In addition, various limitations in dental practice also become barriers in providing care [9, 10, 21, 22, 25]. As a result, in this study, the barrier factor was incorporated into the research model to examine if the predictive value of the intention and practice factors in providing preventive care increased. The discovery of these determinants is likely to narrow the gap between dentists' intentions and preventive behaviors.

The exploratory model examined in this study suggests that barriers will improve the predictive value of dentists' real preventive practice outside of the initial TPB construct [19]. This is in accordance with the limitations of the original TPB model, which assumes that individuals always have opportunities and resources to perform a behavior. Whereas, in reality, many other factors are also influential [10, 23]. Moreover, in the context of this study, dentists do not have complete control over providing treatments [21, 22]. This is also consistent with the elimination of the education variable (EDU) of the intention in one of the items referred to in preventive practices in this model. This could be due to the fact that educational measures are the most basic preventative practices, requiring no additional costs from patients [7]. So, most of the dentists will not skip oral hygiene instruction during everyday practice.

According to this TPB extension model, the TPB construct can describe 55.5% of the dentist's intention, while the addition of barriers from multiple sectors can duplicate the predictive value of preventive practice from 7.6% to 17.8%. This implies that, regardless of the dentist's intentions, barriers from the dentist, kid, parents, and health care system are also important in predicting the actual practice [21, 23].

Each variable's partial association was also investigated. There was a significant influence between dental intentions and preventive practice by 18.9%. The greater the dentist's intention, the more preventive practices are carried out. This demonstrates that even when there is a discrepancy between intentions and practice, the dentist's intention still matters and is one of the proximal determinants of the practice [14, 21, 23].

Based on the three independent variables in the TPB construct, only attitude (38.7%) and perception (42.4%) have significant effects on intention. These variables also have a significant indirect effect on preventive practices (7.3% and 8%). This is similar to the results with Yusuf (2015), which showed that only attitudes and perceptions of behavioral control were significant, although in this study, attitudes were stronger predictors [7]. In addition, it is also known that subjective norms have no significant effect on the intentions and preventive practices carried out by dentists. Similar to Dumitrescu (2011), the TPB constructs accurately predict intentions towards oral health behavior, except for the subjective norm [32]. This variable is often found to be weaker predictor of intentions due to the lack of measurement of normative constructs [13].

In this model, only dentists' (12.6%) and parents (19.1%) related barriers had a significant effect on performing preventive practices. This suggests that parents, as the primary caregivers and the ones who give informed consent and make payments, play the most important role in determining whether preventive practices will be performed on their children [10, 21, 23]. Although not significant, barriers from the health care system that can explain 9.9% of preventive practices still needs to be considered.

Thus, despite the need for careful interpretation due to the limits of the study, there are several key points that is learned from this study. Using an interpersonal approach, TPB is still a relevant theory, providing a conceptual framework regarding the intention to provide certain practices. However, there's a large gap between intention and practice that needs to be considered when formulating a health plan and policy. Zooming out the problem and seeing multiple stakeholders involved is the first step to identify proper actions.

Based on the findings in this study, efforts to reduce the prevalence of caries in children are a complex process that involves all parties, from the government as a regulator of the health care system, dentists as health care providers, parents as the main caregivers, and the child patients themselves [9, 10, 22, 33]. Implications for government policy can be in the form of covered-preventive practices by national insurance and facilitating health service with preventive materials so that more people will have access to this treatment. Government-owned health services should further facilitate dentists' conduct of preventive practices by providing sufficient resources and appropriate remuneration to increase dentist motivation so no gap and inequalities between government and private practices and dentists practicing in rural or urban area [32].

In terms of dentists themselves, education, both formal and further continuing education, plays an important role in improving attitudes, perceptions of behavioral control, and dentists' intentions to carry out preventive practices, so that dentists' motivation to carry out preventive practices can be higher. Education regarding preventive practices, especially silver diamine fluoride application, needs to be intensified because there are still many dentists who do not know about it [34]. Changes in dentists' perceptions, where some still consider preventive dental care to have a lower priority than other treatments, need to be done [35]. Most of the dentists that participate in this study is having less than 10 years experience, means the results may not be representative to older dentist with longer experience. However, more recent shifts focusing more on prevention in dental academic curriculum may have made this study more relevant to current and future situations [24, 34].

It cannot be denied that although this study looked at the dentist's point of view, pediatric patients and their parents are the key factors to reduce caries prevalence in early ages and over the life-course [35, 36]. Based on this study, it was found that the barriers from the side of pediatric patients were basically lower than the barriers to curative practices explored in previous studies [21, 37]. This needs to be a signal that preventive is indeed easier than curative, so this kind of treatment needs to be the main focus as early as possible [38]. Exposure to early oral care also creates positive perceptions that can increase awareness of good oral health and maximize the utilization of oral health services [37]. Oral health education for parents and prospective parents remains an important task and the main target of oral health programs for young children, so that this knowledge can increase healthy oral health behavior and the utilization of oral health services [35].

This study has several limitations. Snowball sampling, a non-probabability sampling method, that employed in this study may produce less accurate findings that cannot be generalized to the full population. Only dentists who are informed about preventive care may agree to participate in this study as a result of the sampling method. There is also no information on the number of dentists who were refused from participating in this study, and the response rate cannot be estimated due to the nature of the sampling technique. A sample size that is undistributed evenly across different provinces may result in less reliable results; thus larger and more proportional sample size for further studies may improve the outcome. However, despite the unproportional demographic distribution of the samples, most of the respondents were from Java Island and worked in the private sector, which aligns with Indonesian previous healthcare spatial analysis [39].

There is a possibility of measurement and social desirability bias because this online survey is self-administered, so the researcher cannot really know whether the respondent answered honestly or if there were other factors that influenced the respondent's answer [40]. In addition, some questions related to the number of patients and the number of practices carried out may not correspond to actual conditions due to the limitations of this study, where data collection was only done at one time. Independent variable is only measured based on their beliefs without actual evaluation, which may provide inaccurate answers. Future research should obtain a bigger sample size and assess other variables from IBM, such as knowledge and skills, salience, environmental constraints, and habit to obtain bigger predictive value to preventive practices [20]. Longitudinal studies may benefit to provide accurate measurements of the practice. Also, assessing other parties point-of-view that involved will be beneficial and show other perspectives. Such research could show a better understanding of multiple factors influencing health preventive practices.

## Conclusion

The extended version of theory of planned behavior is a fit and relevant model, explaining 55.5% of dentists' intentions to undertake preventive procedures and 18% of preventive practices. Compared to the original one, this model describes dentists’ preventive behaviors better. Perceived behavioral control was the most powerful predictor of intention (44.2%) and practice (8.8%), while parental barrier had the highest effect influencing dentists' preventive care (18.7%). Each stakeholder barriers should be addressed through oral health programs and policies, and dentists must be taught to overcome these barriers (through formal or continuing education) in order to maximize caries prevention strategies.

## Data Availability

The raw data are not publicly available due to ethical restrictions but are available from the corresponding author to any author who wishes to collaborate with us.

## References

[1] Watt RG, Mathur MR, Aida J, Bönecker M, Venturelli R, Gansky SA (2018). Oral health disparities in children: a canary in the coalmine?. Pediatr Clin North Am.

[2] Policy on Early Childhood Caries (ECC) (2017). Classifications, consequences, and preventive strategies. Pediatr Dent.

[3] Chen J, Duangthip D, Gao SS (2021). Oral health policies to tackle the burden of early childhood caries: a review of 14 countres/regions. Front Oral Health.

[4] Pierce A, Singh S, Lee J, Grant C, Cruz de Jesus V, Schroth RJ (2019). The burden of early childhood caries in canadian children and associated risk factors. Front Public Health.

[5] Tinanoff N, Baez RJ, Diaz Guillory C (2019). Early childhood caries epidemiology, aetiology, risk assessment, societal burden, management, education, and policy: global perspective. Int J Paediatr Dent.

[6] Dima S, Chang WJ, Chen JW, Teng NC (2018). PM31_early childhood caries-related knowledge, attitude, and practice: discordance between pediatricians and dentists toward medical office-based prevention in Taiwan. Int J Environ Res Public Health.

[7] Yusuf H, Kolliakou A, Ntouva A, Murphy M, Newton T, Tsakos G, Watt RG (2015). Predictors of dentists’ behaviours in delivering prevention in primary dental care in England: using the theory of planned behaviour. BMC Health Serv Res.

[8] Baâdoudi F, van Exel JNA, Ali FM, Maskrey N, van der Heijden GJMG, Duijster D (2019). Perspectives of general dental practitioners on preventive, patient-centred, and evidence-based oral healthcare—A Q-methodology study. Arora A, ed. PLOS ONE.

[9] Rosing K, Leggett H, Csikar J (2019). Barriers and facilitators for prevention in Danish dental care. Acta Odontol Scand.

[10] Suga USG, Terada RSS, Ubaldini ALM (2014). Factors that drive dentists towards or away from dental caries preventive measures: systematic review and metasummary. Wen Z, ed. PLoS ONE.

[11] Akbar AA, Al-Sumait N, Al-Yahya H, Sabti MY, Qudeimat MA (2018). Knowledge, attitude, and barriers to fluoride application as a preventive measure among oral health care providers. Int J Dent.

[12] Rani T, Reddy Er, Merum K, Srujana M, Raju Ss, Seth M (2020). General dentists’ knowledge, attitude, and practice guidelines toward pediatric dentistry. CHRISMED J Health Res.

[13] Armitage CJ, Conner M (2001). Efficacy of the theory of planned behaviour: a meta-analytic review. Br J Soc Psychol.

[14] Bosnjak M, Ajzen I, Schmidt P (2020). The theory of planned behavior: Selected recent advances and applications. Eur J Psychol.

[15] Ajzen I (2020). The theory of planned behavior: frequently asked questions. Hum Behav Emerg Technol.

[16] Bonetti D, Johnston M, Clarkson JE, Grimshaw J, Pitts NB, Eccles M, Steen N, Thomas R, Maclennan G, Glidewell L, Walker A (2010). Applying psychological theories to evidence-based clinical practice: identifying factors predictive of placing preventive fissure sealants. Implement Sci.

[17] Lihua D (2022). An extended model of the theory of planned behavior: an empirical study of entrepreneurial intention and entrepreneurial behavior in college students. Front Psychol.

[18] Sheikh V, Barati M, Khazaei S, Jormand H (2022). Factors related to treatment adherence behaviors among old-age hemodialysis patients in Hamadan, Iran: the application of the extended theory of planned behavior during Covid-19 pandemic. BMC Nephrol.

[19] Shi H, Wang J, Huang R, Zhao J, Zhang Y, Jiang N, Tanimoto T, Ozaki A, Shao C, Wang J, He X, Xu X (2021). Application of the extended theory of planned behavior to understand Chinese students' intention to improve their oral health behaviors: a cross-sectional study. BMC Public Health.

[20] Montano D, Kasprzyk D (2015). Theory of reasoned action theory of planned behavior, and the integrated behavioral model. Health Behav.

[21] Lee GH, McGrath C, Yiu CK (2015). Barriers to providing oral health care to pre-school children-differences between paediatric dentists' and general dental practitioners' beliefs. Community Dent Health.

[22] Nagarajappa R, Sanadhya S, Batra M, Daryani H, Ramesh G, Aapaliya P (2015). Perceived barriers to the provision of preventive care among dentists of Udaipur. India J Clin Exp Dent.

[23] Leggett H, Csikar J, Vinall-Collier K, Douglas GVA (2021). Whose responsibility is it anyway? exploring barriers to prevention of oral diseases across Europe. JDR Clin Transl Res.

[24] Khairinisa S, Setiawati F, Darwita RR, Maharani DA. Perceived Barriers among Indonesian General Dentists in Providing Caries Preventive Care for Pediatric Patients. Eur J Dent. 2023 Aug 17. 10.1055/s-0043-1771336 PMC11132772 37591284

[25] Arheiam A, Masoud I, Bernabé E (2014). Perceived barriers to preventive dental care among Libyan dentists. Libyan J Med.

[26] Gjersing L, Caplehorn JR, Clausen T (2010). Cross-cultural adaptation of research instruments: language, setting, time and statistical considerations. BMC Med Res Methodol.

[27] Utami M, Setiawati F, Ahmad MS, Adiatman M (2022). Cross-cultural adaptation and psychometric properties of the Indonesian version of theory of planned behavior questionnaire to measure dental attendance of children with hearing loss: a pilot study. Spec Care Dentist.

[28] Hair JF, Hult GTM, Ringle CM, Sarstedt M, Danks NP, Ray S. Partial Least Squares Structural Equation Modeling (PLS-SEM) Using R: A Workbook. Springer International Publishing; 2021. 10.1007/978-3-030-80519-7.

[29] Hult GTM, Sarstedt M, Ringle CM. A Primer on Partial Least Squares Structural Equation Modeling (PLS-SEM). Sage Publication, Inc; 2022.

[30] Yokoyama Y, Kakudate N, Sumida F, Matsumoto Y, Gilbert GH, Gordan VV (2013). Dentists’ practice patterns regarding caries prevention: results from a dental practice-based research network. BMJ Open.

[31] Tan SHX, Vernazza CR, Nair R (2017). Critical review of willingness to pay for clinical oral health interventions. J Dent.

[32] Dumitrescu AL, Wagle M, Dogaru BC, Manolescu B (2011). Modeling the theory of planned behavior for intention to improve oral health behaviors: the impact of attitudes, knowledge, and current behavior. J Oral Sci.

[33] Pakdaman A, Yarahmadi Z, Kharazifard MJ (2015). Self-reported knowledge and attitude of dentists towards prescription of fluoride. J Dent Tehran Iran.

[34] Yusuf H, Tsakos G, Ntouva A (2015). Differences by age and sex in general dental practitioners’ knowledge, attitudes and behaviours in delivering prevention. Br Dent J.

[35] Khodadadi E, Niknahad A, Naghibi Sistani MM, Motallebnejad M (2016). Parents’ oral health literacy and its impact on their children’s dental health status. Electron Physician.

[36] Ellakany P, Madi M, Fouda SM, Ibrahim M, AlHumaid J (2021). the effect of parental education and socioeconomic status on dental caries among saudi children. Int J Environ Res Public Health.

[37] Duangthip D, Chen K, Gao S, Lo E, Chu C (2017). Managing early childhood caries with atraumatic restorative treatment and topical silver and fluoride agents. Int J Environ Res Public Health.

[38] Basir L, Rasteh B, Montazeri A, Araban M (2017). Four-level evaluation of health promotion intervention for preventing early childhood caries: a randomized controlled trial. BMC Public Health.

[39] Gofur NRP, Aghasy AZZ, Gofur ARP (2021). Spatial distribution analysis of dentists, dental technicians, and dental therapists in Indonesia. F1000Research.

[40] Badejo MA, Ramtin S, Rossano A, Ring D, Koenig K, Crijns TJ (2022). Does adjusting for social desirability reduce ceiling effects and increase variation of patient-reported experience measures?. J Patient Exp.

